# Cardamonin Reduces Acetaminophen-Induced Acute Liver Injury in Mice via Activating Autophagy and NFE2L2 Signaling

**DOI:** 10.3389/fphar.2020.601716

**Published:** 2020-11-13

**Authors:** Qiushi Xu, Yunhui Fan, Juan J. Loor, Yusheng Liang, Xudong Sun, Hongdou Jia, Chenxu Zhao, Chuang Xu

**Affiliations:** ^1^ College of Animal Science and Veterinary Medicine, Heilongjiang Bayi Agricultural University, Daqing, China; ^2^ Mammalian NutriPhysioGenomics, Department of Animal Sciences and Division of Nutritional Sciences, University of Illinois, Urbana, IL, United States

**Keywords:** cardamonin, acetaminophen, acute liver injury, autophagy, NFE2L2

## Abstract

Cardamonin (CD), a naturally occurring chalcone derived from the *Alpinia* species, has been shown to exert antioxidant and anti-inflammatory activity, but its role in the prevention of acetaminophen- (APAP-) induced hepatotoxicity remains elusive. The objective of this study was to determine the protective effects of CD against APAP-induced acute liver injury (ALI) and the underlying mechanisms. Wild-type or transcription factor nuclear factor erythroid 2-related factor 2- (NFE2L2-) deficient mice were treated with CD (50 or 100 mg/kg, i.p.) or vehicle for 24 h. Subsequently, these mice were challenged with APAP (400 mg/kg, i.p.) for 6 h. Liver and blood samples were collected to evaluate liver injury and protein abundance. Treatment with CD significantly reduced APAP-induced hepatotoxicity. Furthermore, CD effectively reduced APAP-induced inflammation by inhibiting high mobility group box 1 (HMGB1), toll-like receptor 4 (TLR4), and NOD-like receptor protein 3 (NLRP3) signaling. In addition, CD induced activation of sequestosome 1 (p62) and NFE2L2 signaling and facilitated autophagy. By applying autophagy inhibitor 3-methyladenine (3-MA; 20 mg/kg, i.p.), further mechanistic exploration revealed that NFE2L2 deficiency promoted autophagic activity induced by CD treatment, which was conducive to the hepatoprotective effect of CD against APAP-induced hepatoxicity in NFE2L2^−/−^ mice. Overall, data suggest that CD has hepatoprotective effect against APAP-induced ALI, which might contribute to the activation of NFE2L2 and autophagy.

## Introduction

Acetaminophen (N-acetyl-p-aminophenol; APAP) is a ubiquitous and widely used nonprescription drug for the relief of pain and fever (Bessems and Vermeulen, 2001). An important side-effect of APAP is dose-dependent hepatotoxicity. Acute liver injury (ALI) caused by drug toxicity, particularly due to accidental or intentional intake of an overdose of APAP, is the main causal agent for ALI worldwide, accounting for approximately 50% of all cases (Ostapowicz et al., 2002; Gunawan and Kaplowitz, 2007). Liver injury disorders caused by excessive administration of APAP are accompanied by sterile inflammation and oxidative stress, which further exacerbates liver injury (Sanz-Garcia et al., 2013; Du et al., 2016). Thus, investigating novel and efficient protective agents against sterile inflammatory responses that also strengthen antioxidant defenses in ALI is warranted.

It is well known that APAP poisoning generates excess N-acetyl-p-benzoquinone imine, which evokes depletion of glutathione (GSH) and triggers oxidative stress that ultimately results in hepatocellular death (Mitchell et al., 1973; Jaeschke et al., 2012). Extensive cell necrosis caused by APAP-induced hepatotoxicity leads to release of cellular contents and a marked increased blood serum of enzymes related to liver function, which further activates various pattern recognition receptors (PRRs). Toll-like receptors (TLRs) are a major class of PRRs that regulate intracellular inflammation by interaction with insult components (Akira et al., 2001). The damage-associated molecular patterns (DAMPs) released by cells subsequently trigger innate immune systems via TLR4-dependent induction of proinflammatory cytokines and transcription of the NOD-like receptor protein 3 (NLRP3) inflammasome pathway. Activation of inflammasome signaling can induce the cleaved proforms of certain interleukins (ILs) and cytokines (Kubes and Mehal, 2012). In addition, during APAP hepatotoxicity, high mobility group box 1 (HMGB1) is identified as a key extracellular coordinator by promoting the inflammation in ALI (Antoine et al., 2010) and its concentration in serum correlates with progression and prognosis of ALI in humans (Antoine et al., 2012).

Among various mechanisms involved in antioxidant responses, activity of the transcription factor nuclear factor erythroid 2-related factor 2 (NFE2L2) is crucial for ameliorating various inflammation- and oxidative stress-related disorders (Kim et al., 2010). NFE2L2 is structurally held in the cytosol and binding to its main inhibitor, kelch-like ECH-associated protein 1 (Keap1), under basal conditions. Under oxidative stress, NFE2L2 segregates from Keap1 binding and translocates to the nucleus and activates heme oxygenase-1 (HO-1), superoxide dismutase 1 (SOD1), catalase (CAT), and glutathione-S-transferase (GST), which are regarded as downstream antioxidant target genes of NFE2L2 (Fisher et al., 2013). Despite the fact that activation of NFE2L2 not only exerts a protective effect against oxidative stress, but rescues the organism from inflammation in the development of liver injury (Dong et al., 2009), the role of NFE2L2 pathway as a target for the treatment of ALI in humans is unclear.

In eukaryotic cells, autophagy is a highly conserved homeostatic mechanism that degrades damaged organelles or cytoplasmic constituents via lysosomal machinery (Boya et al., 2013). Autophagy is a key regulator of cell homeostasis and survival, while increased inflammatory responses and oxidative stress that result from a deficiency in autophagy ultimately lead to pathological conditions in various tissues especially in liver (Rani et al., 2016). In macrophages, previous studies indicate that there is an inverse relationship between activation of autophagy and NLRP3 inflammasomes (Liu et al., 2018a). Furthermore, autophagy may inhibit production of proinflammatory cytokines via suppressing the NLRP3 inflammasome activation in ALI (Han et al., 2016). Therefore, therapeutic strategies for reversing autophagy dysfunction could have beneficial effects in treatment of ALI.

Cardamonin (2′,4′-dihydroxy-6′-methoxychalcone, CD) is a naturally occurring chalcone derived from the *Alpinia* species (zingiberaceous plant species) that can exert anti-inflammatory and antioxidant activity (Gonçalves et al., 2014). For instance, by reducing oxidative stress and inflammation, CD ameliorated functional and structural damage of the liver following ischemia-reperfusion (Atef et al., 2017). In addition, CD can serve as an anticancer agent through enhancing autophagy via the mechanistic target of rapamycin (mTOR) suppression (Settembre et al., 2011). To our knowledge, there have been no evaluations of the potential for CD to elicit a protective effect on APAP-induced ALI. Thus, the objectives of this study were to investigate the protective effects of CD on liver injury induced by APAP overdose and explore the underlying mechanisms including NFE2L2 and autophagy signaling pathways.

## Materials and Methods

### Chemicals and Reagents

Cardamonin (CD), 98% pure, was from Sigma-Aldrich (cat. no. C8249; St. Louis, MO, United States). APAP and dimethyl sulfoxide were obtained from Sigma-Aldrich (St. Louis, MO, United States). Antibodies against Beclin1 (1:1,000; cat. no. 11306-1-AP), autophagy related 5 (Atg5; 1:500; cat. no. 10181-2-AP), Atg7 (1:5,000; cat. no. 67341-1-Ig), microtubule-associated protein light chain 3 (LC3; 1:500; cat. no. 18725-1-AP), NOD-like receptor protein 3 (NLRP3; 1:500; cat. no. 19771-1-AP), the transcription factor EB (TFEB; 1:1,000; cat. no. 13372-1-AP), mitogen-activated protein kinase (p38 MAPK; 1:2,000; cat. no. 66234-1-Ig), Keap1 (1:2,000, cat. no. 10503-2-AP), and HMOX-1 (1:1,000, cat. no. 10701-1-AP) were obtained from Proteintech (Wuhan, Hubei, China), and cleaved-caspase-1 (1:1,000; cat. no. #89332), caspase-1 (1:1,000; cat. no. #24232), cleaved-IL-1β (1:1,000; cat. no. #63124), IL-1β (1:1,000; cat. no. #31202), phosphorylated (p)-p38 MAPK (1:1,000; cat. no. #4511), and phosphorylation of c-Jun N-terminal kinase (p-JNK; 1:2,000; cat. no. #9255)/JNK (1:1,000; cat. no. #9252) were obtained from Cell Signaling Technology, Inc. (Danvers, MA, United States). Antibodies against NFE2L2 (1:1,000; cat. no. Ab89443), AMP-activated protein kinase (AMPK; 1 μg/ml; cat. no. Ab80039), phosphorylated (p)-AMPK (1:1,000; cat. no. Ab23875), phosphorylated sequestosome 1 (p-p62; 1:1,000; cat. no. Ab211324), GST (1:1,000; cat. no. Ab111947), p-mTOR (1:2,000; cat. no. Ab109268), β-actin (1:2,000; cat. no. Ab8226), and Lamin B1 (1:5,000, Ab194109) were from Abcam (Cambridge, MA, United States). Additionally, alanine transaminase (ALT; cat. no. C009-3-1), aspartate aminotransferase (AST; cat. no. C010-3-1), malondialdehyde (MDA; cat. no. A003-1-2), reactive oxygen species (ROS; cat. no. E004-1-1), GSH (cat. no. A005-1-2), and superoxide dismutase (SOD; cat. no. A001-3-2) test kits were obtained from Nanjing Jiancheng Bioengineering Institute (Nanjing, China).

### Animals and Experimental Design

Wild-type (WT) and NFE2L2-deficient (NFE2L2^−/−^) C57BL/6 male mice weighing 18–22 g, 6–8 weeks old, were purchased from Cyagen Biosciences Inc. (Certificate No. KOAIP190930WZ1; Guangzhou, China). All animals were raised under SPF conditions. All animal procedures were performed in accordance with the Guidelines for the Care and Use of Experimental Animals in Heilongjiang Bayi Agricultural University. The Animal Ethics Committee of Heilongjiang Bayi Agricultural University (Daqing, China) approved the study protocol.

Drug-induced liver injury was caused by intraperitoneal injection of APAP as described previously (Lv et al., 2018). To evaluate the hepatoprotective effects of CD on APAP-induced liver injury, WT mice were randomly divided into five groups (*n* = 10 mice per group): control (PBS), CD (50 mg/kg) alone group, APAP (900 mg/kg) alone group, and CD (50 or 100 mg/kg) + APAP group. Briefly, mice were injected intraperitoneally with CD (50 or 100 mg/kg) for 24 h followed by treatment with APAP (900 mg/kg). To investigate the participation of NFE2L2 in the hepatoprotective effect of CD in APAP-induced ALI, WT or NFE2L2^−/−^ mice were divided into four groups (*n* = 10 mice per group): control, CD (100 mg/kg) alone group, APAP (900 mg/kg) group, and CD (100 mg/kg) + APAP (900 mg/kg) group. To further verify the role of autophagy and the therapeutical effect of CD treatment in ALI, mice were intraperitoneally injected with autophagy inhibitor 3-methyladenine (3-MA) for 2 h before CD treatment. WT or NFE2L2^−/−^ mice were divided into five groups (*n* = 10 mice per group): control, CD (100 mg/kg) alone group, APAP (900 mg/kg) group, CD (100 mg/kg) + APAP (900 mg/kg) group, 3-MA (20 mg/kg) + APAP (900 mg/kg) group, and 3-MA (20 mg/kg) + CD (100 mg/kg) + APAP (900 mg/kg) group. After 6 h of APAP administration, mice were euthanized by cervical dislocation and liver tissue and serum harvested for biomarker profiling, histopathological evaluation, ELISA, or western blot assays.

### Histopathological Evaluation

A subsample of liver tissue was fixed immediately with 10% buffered formalin and then embedded in paraffin. Tissue was cut into a thickness of 5 μm and pathological changes were evaluated by hematoxylin-eosin (H&E) staining using light microscopy. Histological changes were evaluated by an ordinal scale for ranking the severity of hepatic injury in accordance with a protocol described previously (Lv et al., 2019). Liver damage of stained sections was graded in a 4-point scale as follows: stage 1 = no damage; stage 2 = mild damage; stage 3 = moderate damage; stage 4 = severe damage.

### Assessment of Liver Function and Oxidative Stress

All mice were sacrificed 6 h after APAP exposure and liver and blood serum collected for biochemical analyses. Serum and liver activities of ALT and AST were measured using commercial kits according to the manufacturer's instructions. In addition, liver tissue was homogenized and dissolved in extraction buffer to analyze concentrations of MDA, ROS, SOD, and GSH according to manufacturer’s instructions. All results were normalized by total protein concentration in each sample.

### Inflammation Biomarkers Analysis Using Immunosorbent Assay (ELISA)

Blood sample was obtained from each mice and serum harvested for evaluation of the tumor necrosis factor alpha (TNF-α; cat. no. SMTA00B), IL-1β (cat. no. SMLB00C), and IL-6 (cat. no. SM6000B) concentrations using ELISA kits following manufacturer’s instructions (R&D Systems, Minneapolis, MN, United States).

### Western Blot Analysis

Total protein or nuclear protein was extracted from fresh liver tissue using protein extraction kits according to the manufacturer’s protocol (cat. no. W034-1-1 and W037-1-1, respectively; Nanjing Jiancheng Bioengineering Institute). Protein concentrations were determined by the BCA protein assay. A total of 20 μg of protein from each sample was separated by a 12% SDS-PAGE and transferred onto 0.45 μm polyvinylidene difluoride membrane. The polyvinylidene difluoride membrane was blocked with 5% (w/v) nonfat milk for 2 h followed by incubation with primary antibodies and secondary antibodies. The membrane was then washed with TBST. Lastly, the membranes were visualized with the enhanced chemiluminescence solution in western blotting detection system in accordance with manufacturer’s instruction. Band intensities were quantified using ImageJ gel analysis software (National Institutes of Health, Bethesda, MD, United States). All experiments were performed in triplicate.

### Statistical Analysis

Data are expressed as the mean ± SEM. Statistical analysis was conducted using SPSS software version 25.0 (IBM, Chicago, IL) and GraphPad Prism program (Prism 8.3.0; GraphPad Software, San Diego, CA). Comparisons between groups were conducted using one-way ANOVA, whereas multiple comparisons were based on the ANOVA (LSD method). A *p* value < 0.05 was considered statistically significant and a *p* value < 0.01 was considered highly significant.

## Results

### Cardamonin Alleviated Acetaminophen-Induced Acute Liver Injury

**FIGURE 1:**
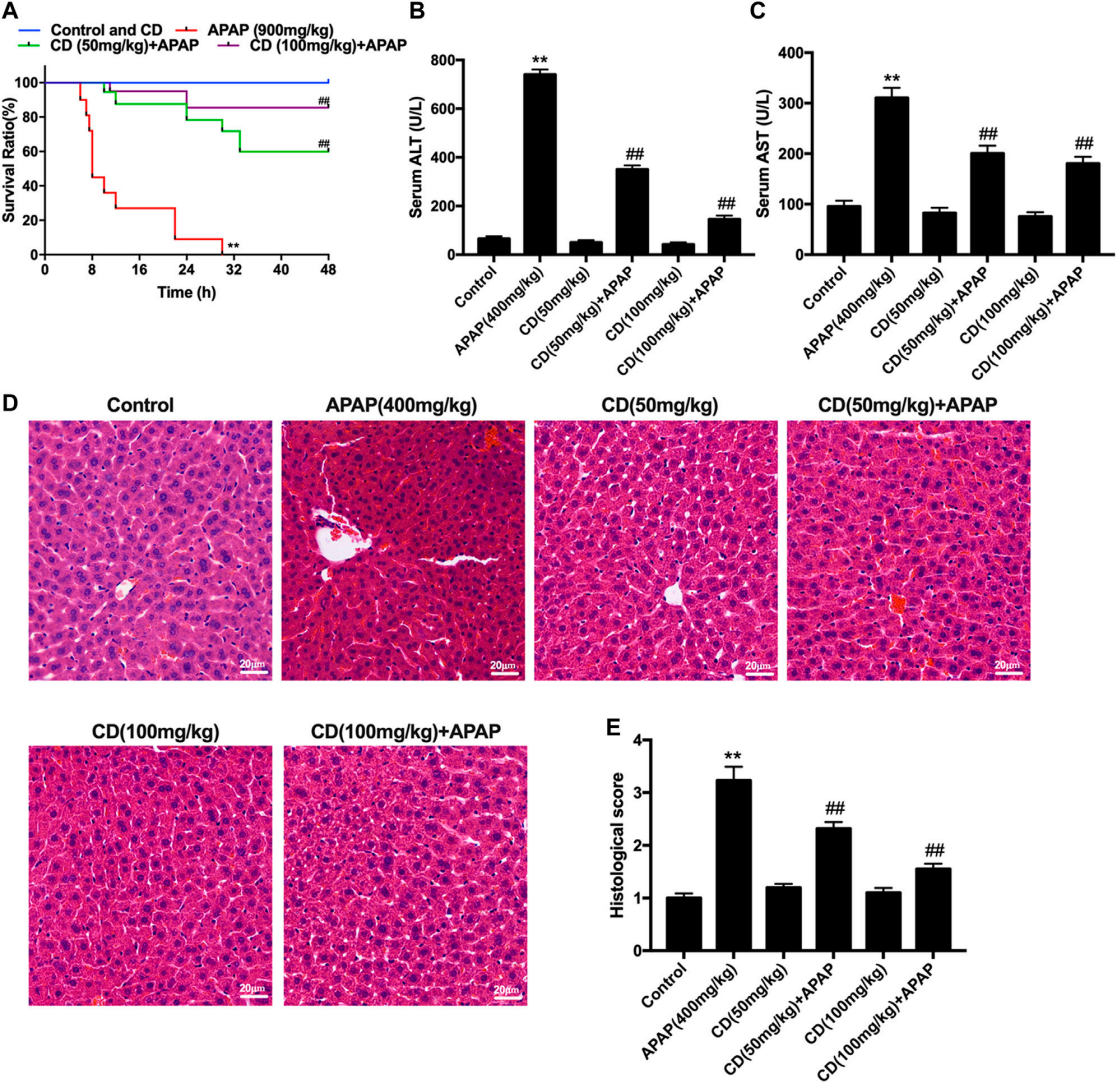
Protective effects of CD on APAP-induced ALI. **(A)** Survival rate of mice within 24 h after APAP administration. **(B,C)** Blood serum was collected for assessment of ALT and AST activities 6 h after injection with APAP. **(D)** Representative histological sections of liver stained with hematoxylin and eosin (H&E) (magnification ×400). **(E)** Liver damage of stained sections was graded in a four-point scale as follows: stage 1 = no damage; stage 2 = mild damage; stage 3 = moderate damage; stage 4 = severe damage. Similar results were obtained from three independent experiments. All data are presented as means ± SEM (*n* = 10 in each group). *p < 0.05 and **p < 0.01 vs. control group; ^#^ *p* < 0.05 and ^##^ *p* < 0.01 vs. APAP group.

Mice in the APAP group began to perish 6 h after APAP challenge with survival rate reaching 0% at 30 h. In contrast, after CD treatment survival rate was dose-dependent and increased to 85% with 100 mg/kg supplementation or to 60% with 50 mg/kg supplementation (Figure 1A). Compared with the APAP group, CD supplementation reduced enzyme activities of ALT and AST in serum (Figures 1B,C). Histological analysis of liver in the APAP group revealed a noticeable disturbance of liver architecture including hemorrhage, hepatocyte necrosis, and neutrophil infiltration, whereas CD treatment alleviated these alterations (Figures 1D,E).

### Cardamonin Inhibits Acetaminophen-Induced Sterile Inflammation Responses in Mice With Liver Injury

**FIGURE 2:**
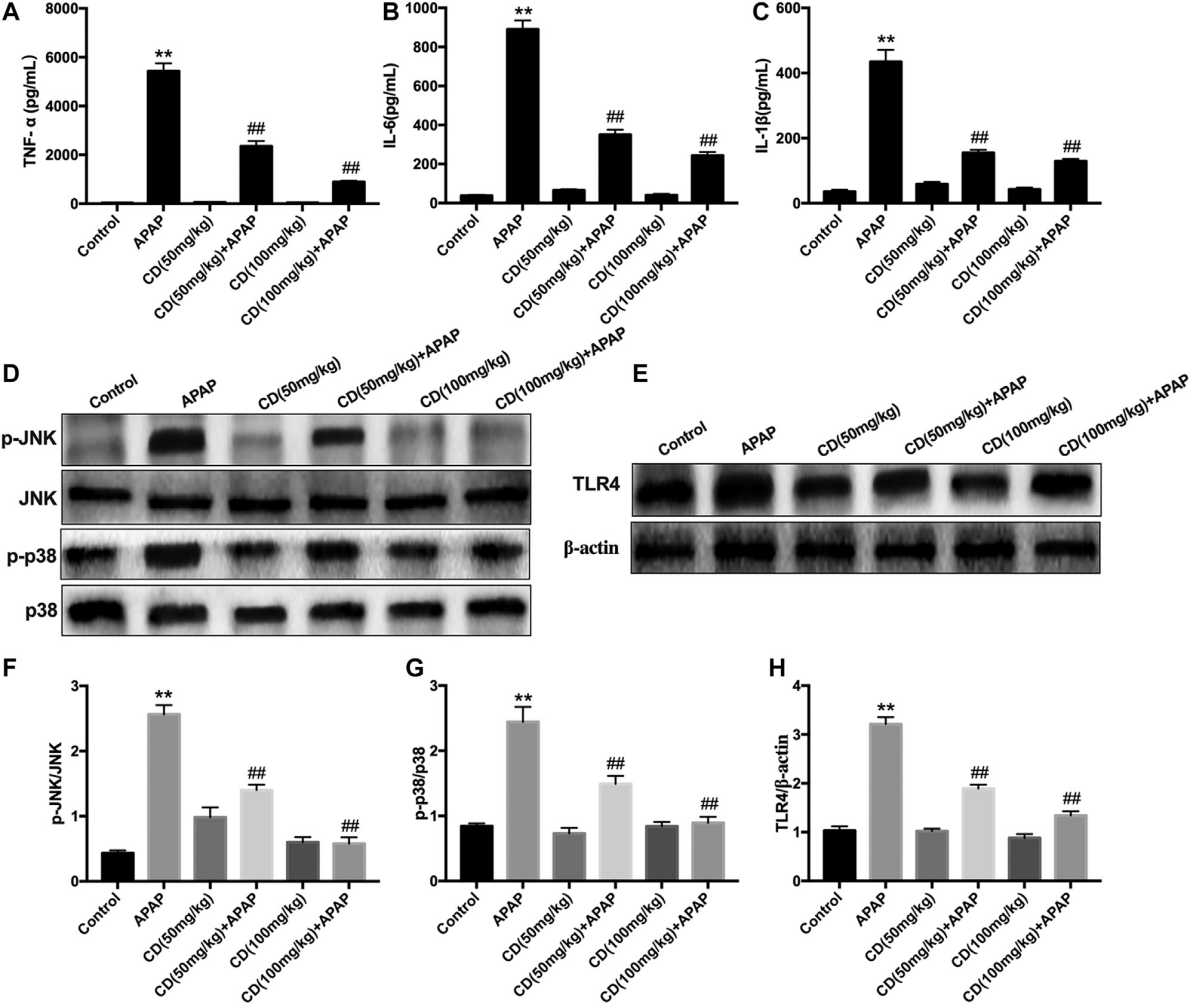
Effect of CD treatment on sterile inflammation in mice with APAP-induced ALI. **(A–C)** Liver tissue collected from the mice 6 h after APAP challenge and used for measurement of TNF-α, IL-6, and IL-1β concentrations. **(D–H)** Effects of CD on the protein abundance of JNK and p38 MAPK in mice with APAP-induced ALI. Similar results were obtained from three independent experiments. All data are presented as means ± SEM (*n* = 10 in each group). *p < 0.05 and **p < 0.01 vs. control group; ^#^ *p* < 0.05 and ^##^ *p* < 0.01 vs. APAP group.

As shown in Figures 2A–C, APAP remarkably stimulated the secretion of TNF-α, IL-6, and IL-1β in serum compared to the control group, whereas CD treatment lessened the production of inflammatory cytokines induced by APAP administration. Compared with the APAP group, CD significantly inhibited the abundance of TLR4 and phosphorylation of JNK and MAPK in APAP-stimulated mice (Figures 2F–H).

### Cardamonin Treatment Inhibits Acetaminophen-Activated NOD-Like Receptor Protein 3 Inflammasome in Acute Liver Injury Mice

**FIGURE 3:**
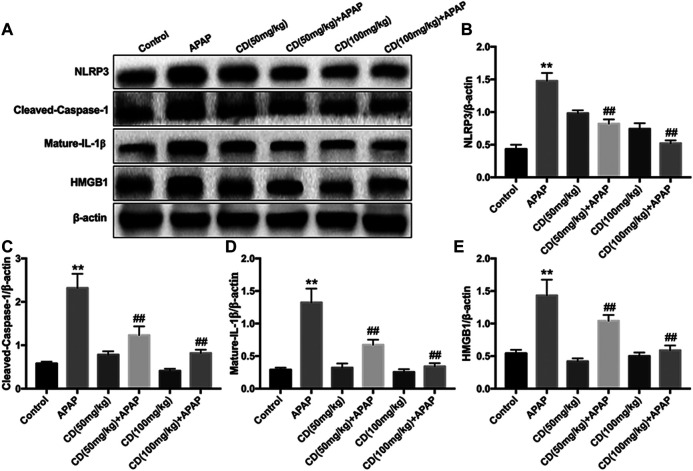
Effect of CD treatment on the APAP-induced NLRP3 inflammasome signaling pathway in ALI mice. Liver tissue was collected 6 h after APAP challenge and analyzed by western blot. **(A–E)** Effects of CD treatment on protein abundance of NLRP3, cleaved-caspase-1, mature-IL-1β, and HMGB1 in mice with APAP-induced ALI. Similar results were obtained from three independent experiments. All data are presented as means ± SEM (*n* = 10 in each group). *p < 0.05 and **p < 0.01 vs. control group; ^#^ *p* < 0.05 and ^##^ *p* < 0.01 vs. APAP group.

Western blot analysis showed that, compared with the control group, the protein abundance of NLRP3, cleaved-caspase-1, mature-IL-1β, and HMGB1 increased in the APAP-treated group. In addition, CD treatment significantly inhibited the activation of NLRP3, cleaved-caspase-1, mature-IL-1β, and HMGB1 in APAP-induced ALI (Figures 3A–E).

### Cardamonin Upregulated Antioxidant Signaling Pathways in Mice With Acetaminophen-Induced Acute Liver Injury

**FIGURE 4:**
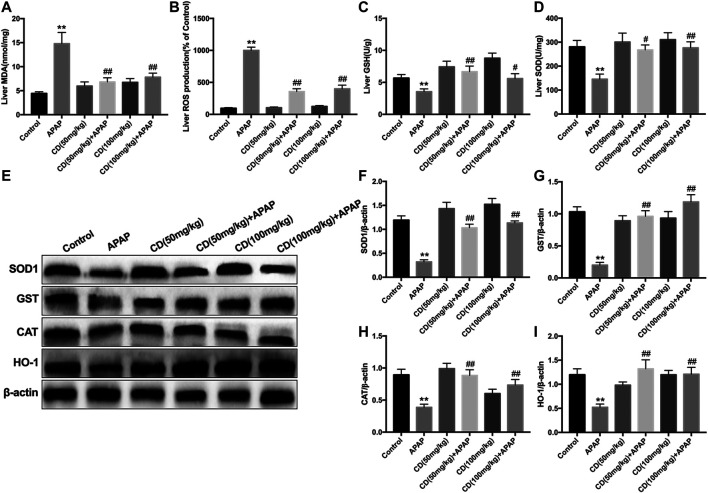
Effects of CD treatment on levels of oxidative markers in APAP-induced ALI. Liver tissue was collected 6 h after APAP injection for assessment of ROS, MDA, SOD, and GSH. **(A–D)** Effect of CD on concentrations of ROS, MDA, SOD, and GSH. **(E–I)** Protein abundance of HMOX1, SOD1, CAT, and GST. β-Actin was used as an internal control. Similar results were obtained from three independent experiments. All data are presented as means ± SEM (*n* = 10 in each group). *p < 0.05 and **p < 0.01 vs. control group; ^#^ *p* < 0.05 and ^##^ *p* < 0.01 vs. APAP group.

Administration of APAP exacerbated the accumulation of MDA and ROS and caused the consumption of GSH and SOD, which might lead to oxidative damage to the liver of mice. However, CD treatment effectively reversed these effects (Figures 4A–D). In addition, the protein abundance of NFE2L2 downstream antioxidant genes, including SOD1, GST, CAT, and HO-1, is consistent with the above results (Figures 4E–I).

### Cardamonin Treatment Upregulated the p62-NFE2L2/Keap1 Signaling Pathway in Mice With Acetaminophen-Induced Acute Liver Injury

**FIGURE 5:**
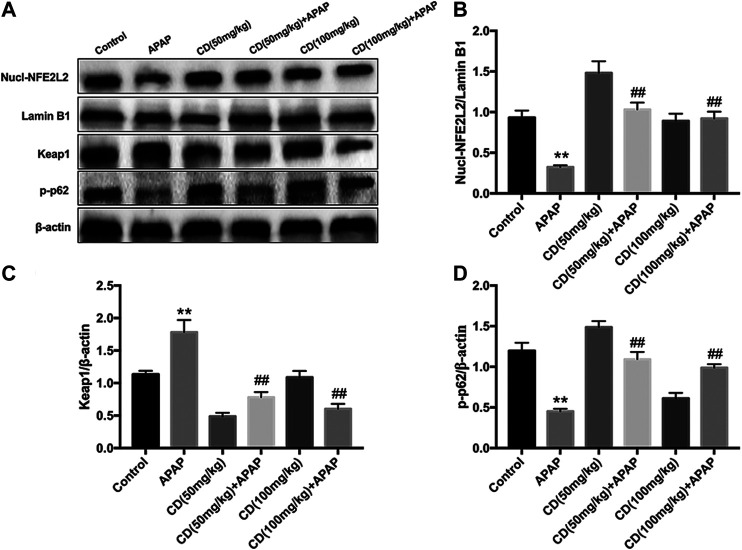
Effects of CD treatment on the p62-NFE2L2/Keap1 signaling pathway in WT mice with APAP-induced ALI. **(A–D)** Liver tissue was collected 6 h after APAP challenge and analyzed by western blot for assessment of nuclear abundance of NFE2L2, protein abundance of Keap1, and the phosphorylation of p62 at ser349. β-Actin or Lamin B1 was used as an internal control. Similar results were obtained from three independent experiments. All data are presented as means ± SEM (*n* = 10 in each group). *p < 0.05 and **p < 0.01 vs. control group; ^#^ *p* < 0.05 and ^##^ *p* < 0.01 vs. APAP group.

Administration of CD effectively promoted nuclear abundance of NFE2L2, whereas Keap1 abundance was downregulated compared to APAP-treated group (Figures 5A–C). In addition, CD treatment significantly enhanced the APAP-induced phosphorylation of p62 in liver injury (Figure 5D).

**FIGURE 6:**
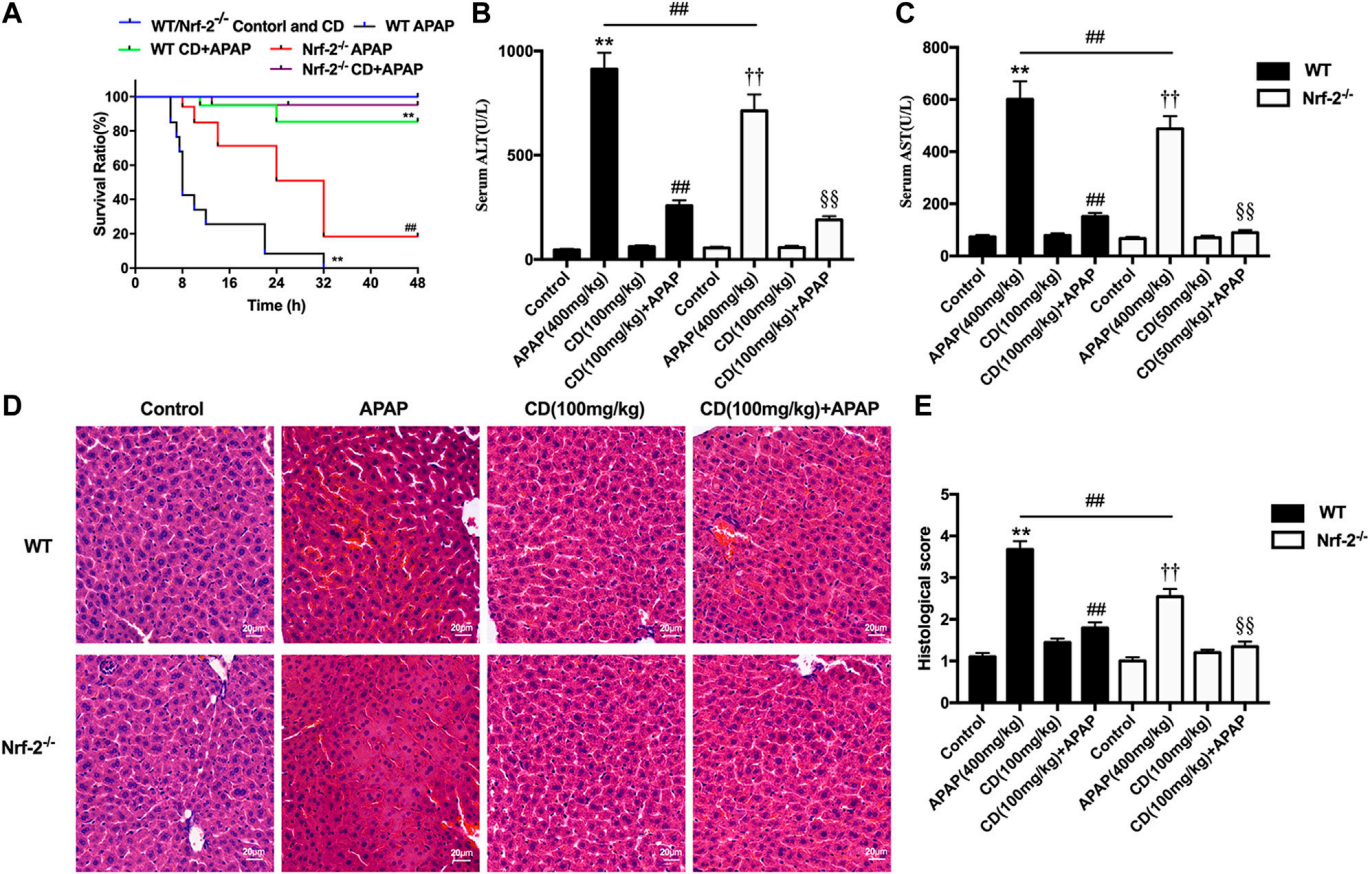
Effects of CD treatment on the p62-NFE2L2/Keap1 signaling pathway in NFE2L2^−/−^ mice with APAP-induced ALI. WT and NFE2L2^−/−^ mice were injected intraperitoneally with CD (100 mg/kg) for 24 h followed by challenge with APAP. **(A)** Survival rate of mice was observed within 24 h after APAP administration. **(B,C)** Blood serum collected at 6 h after injection with APAP for assessment of ALT and AST activities. **(D)** Representative histological sections of liver were stained with hematoxylin and eosin (H&E) (magnification ×400). **(E)** Liver damage of stained sections was graded in a 4-point scale as follows: stage 1 = no damage; stage 2 = mild damage; stage 3 = moderate damage; stage 4 = severe damage. Similar results were obtained from three independent experiments. All data are presented as means ± SEM (*n* = 10 in each group). *p < 0.05 and **p < 0.01 vs. WT control group; ^#^ *p* < 0.05 and ^##^ *p* < 0.01 vs. WT APAP group; ^†^ *p* < 0.05 and ^††^ *p* < 0.01 vs. NFE2L2^−/−^ control group; ^§^ *p* < 0.05 and ^§§^ *p* < 0.01 vs. NFE2L2^−/−^ APAP group.

Survival rates for APAP-treated NFE2L2^−/−^ mice elevated from approximately 20–95% after treatment with CD (Figure 6A). Our results indicate that, in NFE2L2^−/−^ mice, APAP-induced increase of ALT and AST serum levels in WT mice is effectively inhibited (Figures 6B,C). In addition, we observed histopathological changes in WT and NFE2L2^−/−^ mice, suggesting that treatment with CD relieved severe histopathological changes in WT mice and these changes were more significantly alleviated in NFE2L2^−/−^ mice (Figures 6D,E).

### Cardamonin Promotes Autophagy Activation and Attenuates Acetaminophen-Induced Liver Injury

**FIGURE 7:**
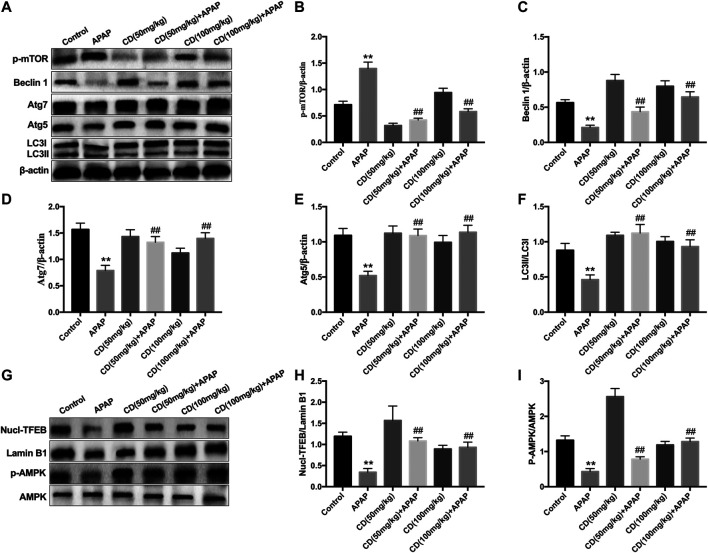
Effect of CD-mediated autophagy on liver injury induced by APAP overdose in mice. Liver tissues were collected from mice 6 h after APAP challenge and analyzed by western blot. **(A–F)** Effects of CD on protein abundance of p62, p-mTOR, Beclin-1, Atg5, Atg7, LC3I, and LC3II measured by western blotting. **(G–I)** Effect of CD on phosphorylation of AMPK and nuclear translocation of TFEB. β-Actin or Lamin B1 was used as an internal control. Similar results were obtained from three independent experiments. All data are presented as means ± SEM (*n* = 10 in each group). *p < 0.05 and **p < 0.01 vs. control group; ^#^ *p* < 0.05 and ^##^ *p* < 0.01 vs. APAP group.

Compared with the control group, the abundance of proautophagy proteins (Beclin1, p62, Atg7, Atg5, and LC3II/LC3I) was reduced in APAP-induced ALI in mice, whereas phosphorylation of mTOR at serine 2,448 was upregulated and the protein abundance of these proautophagy proteins was significantly recovered by CD treatment (Figures 7A–F). Western blot analysis showed that CD led to greater activation of AMPK and enhanced nuclear abundance of TFEB (Figures 7E–I).

### Cardamonin-Induced Autophagy Activation Is Strengthened by NFE2L2 Deficiency in Mice With Acetaminophen-Induced Acute Liver Injury

**FIGURE 8:**
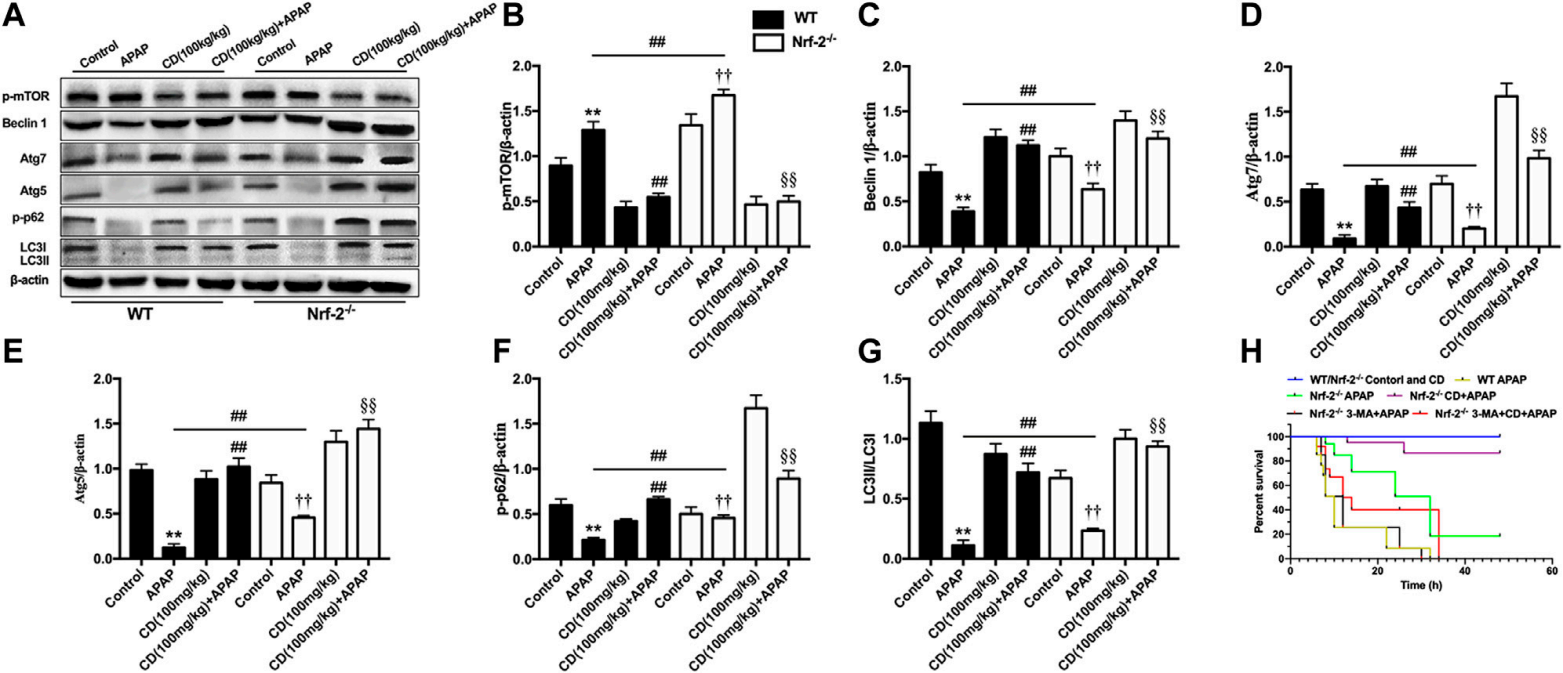
NFE2L2 deficiency strengthened autophagy caused by CD treatment in mice with APAP-induced ALI. WT and NFE2L2^−/−^ mice were injected with CD (100 mg/kg) for 24 h followed by challenge with APAP (900 mg/kg). Liver tissue was collected 6 h after APAP challenge and analyzed by western blotting. **(A–G)** Effects of CD on protein abundance of Beclin-1, Atg5, Atg7, LC3, and phosphorylation of mTOR at serine 2,448 measured by western blot. β-Actin was used as an internal control. Similar results were obtained from three independent experiments. **(H)** Survival rate of mice within 24 h after APAP administration. All data are presented as means ± SEM (*n* = 10 in each group). *p < 0.05 and **p < 0.01 vs. WT control group; ^#^ *p* < 0.05 and ^##^ *p* < 0.01 vs. WT APAP group; ^†^ *p* < 0.05 and ^††^ *p* < 0.01 vs. NFE2L2^−/−^ control group; ^§^ *p* < 0.05 and ^§§^ *p* < 0.01 vs. NFE2L2^−/−^ APAP group.

Compared to WT mice, APAP treatment reduced less abundance of Beclin-1, Atg5, Atg7, and LC3 in NFE2L2^−/−^ mice. CD restored the abundance of proautophagy proteins that reduced by APAP overdose in WT mice, which were found to be significantly strengthened in NFE2L2^−/−^ mice (Figures 8A–G). The survival rate of NFE2L2^−/−^ mice in the APAP group was 19%, whereas the survival rate after 3-MA treatment was 0%. Furthermore, no significant difference was found in the survival rate between APAP-treated WT mice and NFE2L2^−/−^ mice cotreated with 3-MA and CD (Figure 8H).

## Discussion

Acetaminophen-induced liver injury is the most frequently encountered drug-induced hepatotoxicity and remains a major public health problem globally (Kaplowitz, 2005). APAP-induced liver injury depends largely on sterile inflammation and oxidative stress (Yan et al., 2018). Therefore, relieving inflammation and oxidative stress is an effective strategy that can prevent or treat liver injury (Yang et al., 2017). It has been reported that CD has anti-inflammatory and antioxidant effects, which may be closely related to the defense mechanism mediated by the antioxidant pathway encompassing NFE2L2/Keap1 signaling (De Spirt et al., 2016). In this study, we examined the protective effect of CD on APAP-induced liver injury and uncovered for the first time that CD has an effective anti-inflammatory and antioxidant effect against APAP-induced liver toxicity. Based on our data, CD elicits these effects through activation of NFE2L2 signaling and induction of autophagy.

Accumulating evidence indicates that liver injury is accompanied by an increase in ALT and AST in serum and pathological changes in the liver including increased capillary permeability, massive neutrophil infiltration, and release of inflammatory mediators (Lv et al., 2019). Our results indicated that APAP significantly increased mortality, serum activities of ALT and AST, and severity of histopathological changes in the liver. It has been reported that APAP overdose causes liver injury by inducing hepatic sterile inflammation (Cover et al., 2006; Williams et al., 2010). We further evaluated the effect of CD on inflammatory cytokines in serum. Our results showed that CD significantly reduced synthesis of TNF-α, IL-1β, and IL-6 in serum, indicating that CD treatment alleviated the inflammatory responses due to APAP-induced liver injury. It is well accepted that transcriptional activation of proinflammatory cytokines in sterile inflammation induced by APAP overdose is mainly regulated by toll-like receptors and inflammasome activation (Woolbright and Jaeschke, 2017). In addition, the late-stage inflammatory cytokine HMGB1 can stimulate the endogenous inflammatory cascade (Wang et al., 2001). TLR4 can recognize DAMPs released by cells including HMGB1 protein and subsequently induce activation of NLRP3 inflammasome through ROS generation (Jaeschke et al., 2014). Our results showed that CD inhibited APAP-induced oxidative stress and blocked APAP-induced activation of TLR4 and NLRP3 inflammasome in liver tissue. Furthermore, the activation of NLRP3 is closely associated with secretion of mature-IL-1β and cleave-caspase-1, which are closely related to the pathogenesis of liver injury (Kamo et al., 2013; Negash et al., 2013). Our results indicated that CD dramatically inhibited APAP-induced HMGB1, NLRP3, cleave-caspase-1, and mature-IL-1β protein abundance. Thus, we hypothesized that the function of CD to alleviate inflammatory responses and liver injury might be attributed to its antioxidant capacity.

It is well-established that the transcription factor NFE2L2 is a key player in the regulation of antioxidant defense mechanisms against APAP-induced hepatotoxicity (Chan et al., 2001). A previous study revealed that overactivation of NFE2L2/HO-1 signaling inhibited NLRP3 inflammasome, which ameliorated alcohol-induced ALI (Liu et al., 2018b). Activation of NFE2L2 is associated with p62-NFE2L2-Keap1 signaling (Katsuragi et al., 2016), and CD exerted protective effects against oxidative damage through upregulation of NFE2L2-driven antioxidant signaling in neuronal PC12 cells (Peng et al., 2017). Furthermore, NFE2L2 activation is associated with phosphorylation of p62 via competitive disruption of Keap1-NFE2L2 binding (Ichimura et al., 2013). In the present study, we also examined the effect of CD on phosphorylation of p62, and data indicated that CD effectively induced NFE2L2 translocation, p62 phosphorylation at serine 349, and abundance of HO-1 in mice with liver injury induced by APAP overdose. These results indicated that NFE2L2 might play an essential role in protecting against APAP-induced ALI.

Based on the results above, further mechanistic investigations were performed to evaluate whether the hepatoprotective effect of CD was dependent on NFE2L2 by using NFE2L2-deficient mice. In the survival rate analysis, in WT mice the final survival rate was 0% for the APAP group vs. 80% for the CD-treated + APAP group, whereas in the NFE2L2^−/−^ mice it was 20% for the APAP group vs. 95% in the CD-treated + APAP group suggesting that NFE2L2^−/−^ mice were less susceptible to APAP overdose and compared with WT mice were less susceptible to liver damage. Thus, we speculate that CD may mediate other mechanisms to equally or predominantly play a protective role in APAP-induced ALI.

Accumulating evidence to date has revealed that the induction of autophagy could attenuate sterile inflammatory responses by regulating inflammasome activation, indicating an essential role of autophagy in the amelioration of hepatotoxicity induced by APAP (Kurahashi et al., 2016). Furthermore, sustained autophagy could relieve APAP-induced liver injury by modulating oxidative stress (Han et al., 2010; Yan et al., 2018). In the present study, CD treatment induced autophagy via upregulating the protein abundance of Atg3, Atg5, Atg7, Beclin-1, and LC3II conversion all of which were reduced by APAP overdose. The transcription factor EB is crucial in the control of autophagy and its function is partly regulated by mTOR and AMPK signaling (Settembre et al., 2011). Because CD can induce autophagy via suppression of mTOR signaling *in vitro* (Kim et al., 2015; Shi et al., 2018) and the role of TFEB on that process, we sought to investigate the effect of CD on TFEB, mTOR, and AMPK signaling. In the present study, CD enhanced nuclear transcription of TFEB, phosphorylation of AMPK, and inhibited induction of mTOR in APAP-induced ALI. These responses indicated that activation of CD-induced autophagy may be dependent on the AMPK/mTOR signaling pathway.

It is noteworthy that, compared with WT mice, APAP led to reduced abundance of proautophagy proteins in NFE2L2^−/−^ mice and CD treatment upregulated abundance of proautophagy proteins. These responses suggested that NFE2L2 deficiency led to compensatory upregulation of autophagy, which may clarify the higher survival observed and the hepatoprotective effect of CD in NFE2L2^−/−^ mice. In agreement with our results, previous studies have reported that the compensatory activation of autophagy was induced in breast carcinoma cells and cardiomyocytes and was associated with p62-related NFE2L2 activation and inhibition of proteasome activity (Ding et al., 2007; Su and Wang, 2011; Ryoo et al., 2015). To further validate the impact of autophagy in the therapeutical effect of CD treatment in ALI, mice were pretreated with the autophagy inhibitor 3-MA prior to APAP treatment. The results revealed that the pretreatment with 3-MA aggravated liver injury characterized by decreased survival induced by APAP stimulation in NFE2L2-deficient mice. Autophagy inhibitor and CD cotreatment decreased the survival compared with the CD-treated mice in response to APAP challenge, indicating that suppression of autophagy partially reduced the CD-mediated protective effect on APAP-induced ALI in NFE2L2-deficient mice. Collectively, we speculate that both CD-mediated autophagy and upregulation of NFE2L2 improve APAP-induced liver damage, and NFE2L2 deficiency may enhance the compensatory induction of autophagy.

## Conclusion

**FIGURE 9:**
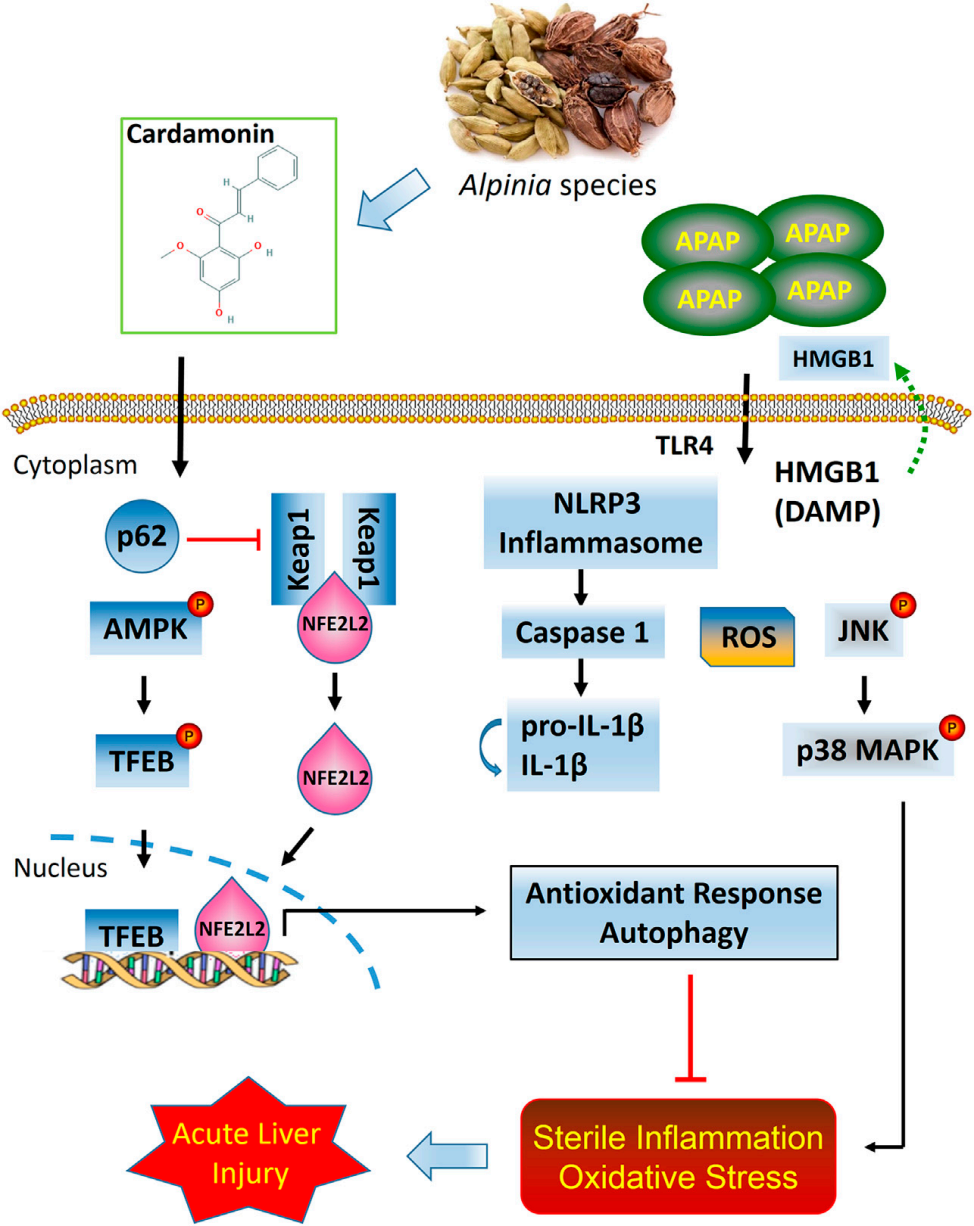
Putative model based on available data of protective effects of CD on APAP-induced ALI.

In summary, the present study provides strong evidence that CD is therapeutically effective in attenuating APAP-induced hepatotoxicity through mechanisms involving activation of NFE2L2 and induction of autophagy. Under conditions of NFE2L2 deficiency, CD can promote compensatory capabilities to enhance autophagic capacity during liver injury. Overall, our data uncovered novel mechanisms for CD in protecting the liver against sterile inflammatory and oxidative damage during APAP-induced ALI (Figure 9).

## Data Availability Statement

The original contributions presented in the study are included in the article/supplementary materials; further inquiries can be directed to the corresponding authors.

## Author Contributions

QX and CX designed research; YF and QX conducted research; QX, XS, HJ, and CZ analyzed data; QX and YF wrote the paper; JL and YL revised the manuscript; CX had primary responsibility for final content. All authors read and approved the final manuscript.

## Funding

This work was supported by the National Key R&D Program of China (Project No. 2017YFD0502200), the National Program on Key Research Project of China (Project 2017YFD0502206), the National Natural Science Foundation of China (Beijing, China; grant no. 31672622).

## Conflict of Interest

The authors declare that the research was conducted in the absence of any commercial or financial relationships that could be construed as a potential conflict of interest.

## Abbreviations

**Abbreviations:** ALI, acute liver injury; ALT, alanine transaminase; AMPK, AMP-activated protein kinase; APAP, acetaminophen; CAT, catalase; CD, cardamonin; DAMPs, damage-associated molecular patterns; GSH, glutathione; GST, glutathione-S-transferase; H&E, hematoxylin-eosin; HMGB1, high mobility group box 1; HO-1, heme oxygenase-1; IL, interleukins; JNK, c-Jun N-terminal kinase; Keap1, Kelch-like ECH-associated protein 1; MAPK, mitogen-activated protein kinase; mTOR, mammalian target of rapamycin; NAPQI, N-acetyl-p-benzoquinone imine; NLR, NOD-like receptor; NLRP3, NOD-like receptor protein 3; NFE2L2, nuclear factor erythroid 2-related factor 2; PRRs, pattern recognition receptors; SOD1, superoxide dismutase 1; TFEB, transcription factor EB; TLRs, toll-like receptors.
